# Cryopreserved human alternatively activated macrophages promote resolution of acetaminophen-induced liver injury in mouse

**DOI:** 10.1038/s41536-025-00393-3

**Published:** 2025-01-22

**Authors:** Maria Elena Candela, Melisande Addison, Rhona Aird, Tak-Yung Man, Jennifer A. Cartwright, Candice Ashmore-Harris, Alastair M. Kilpatrick, Philip J. Starkey Lewis, Anna Drape, Mark Barnett, Donna Mitchell, Colin McLean, Neil McGowan, Marc Turner, James W. Dear, Stuart J. Forbes

**Affiliations:** 1 Centre for Regenerative Medicine, The Institute for Regeneration and Repair, University of Edinburgh, Edinburgh, UK; 2 The Royal (Dick) School of Veterinary Studies and the Roslin Institute, University of Edinburgh, Edinburgh, UK; 3 Scottish National Blood Transfusion Service (SNBTS), The Jack Copland Centre, Heriot-Watt Research Park, Edinburgh, UK; 4 Centre for Precision Cell Therapy for the Liver, Lothian Health Board, Queens Medical Research Institute, Edinburgh, UK; 5 Centre for Cardiovascular Science, University of Edinburgh, The Queen’s Medical Research Institute, Edinburgh, UK

**Keywords:** Liver diseases, Regeneration

## Abstract

Acute liver failure is a rapidly progressing, life-threatening condition most commonly caused by an overdose of acetaminophen (paracetamol). The antidote, N-acetylcysteine (NAC), has limited efficacy when liver injury is established. If acute liver damage is severe, liver failure can rapidly develop with associated high mortality rates. We have previously demonstrated that alternatively, activated macrophages are a potential therapeutic option to reverse acute liver injury in pre-clinical models. In this paper, we present data using cryopreserved human alternatively activated macrophages (hAAMs)—which represent a potential, rapidly available treatment suitable for use in the acute setting. In a mouse model of APAP-induced injury, peripherally injected cryopreserved hAAMs reduced liver necrosis, modulated inflammatory responses, and enhanced liver regeneration. hAAMs were effective even when administered after the therapeutic window for NAC. This cell therapy approach represents a potential treatment for APAP overdose when NAC is ineffective because liver injury is established.

## Introduction

Acetaminophen (paracetamol—APAP) overdose remains the leading cause of acute liver failure (ALF)^1,2^. ALF represents a significant clinical challenge^3^ due to its high mortality and the limited effectiveness of current treatments^4–6^. Factors such as alcohol use, medications, and genetic traits can influence the severity of APAP hepatotoxicity. N-acetylcystein (NAC) is the standard treatment for APAP poisoning^7^, but its efficacy decreases as the time post-ingestion extends^8,9^, indicating the need for additional therapeutic options.

ALF triggered by APAP overdose is characterized by rapid and massive hepatocyte death, leading to liver dysfunction and, potentially, multi-organ failure. It is associated with the systemic inflammatory response syndrome (SIRS), conferring a high risk of multi-organ failure^10–13^. The primary mechanism for liver injury after APAP overdose involves the metabolic conversion of APAP into a toxic metabolite, (N-acetyl-p-benzoquinone imine) NAPQI, which depletes hepatocyte glutathione reserves and causes oxidative damage to cellular proteins^14–16^. This can be combined with an inflammatory cascade, marked by the recruitment of immune cells to the liver and the release of pro-inflammatory cytokines, which can exacerbate tissue damage and promote systemic inflammatory responses^12^.

The APAP mouse model has been used in elucidating the molecular mechanisms of APAP toxicity, highlighting a potential role for the innate immune response in the progression of liver injury. After the injury, the release of pro-inflammatory cytokines and damage-associated molecular patterns (DAMPs), such as High Mobility Group Box 1 (HMGB1)^17^, drives this response. HMGB1, an inflammatory mediator, is released by activated immune cells and translocates from the nucleus to the cytoplasm and eventually to the extracellular space, exacerbating the inflammatory response^17–19^. Alongside the inflammatory response, polymorphonuclear leukocytes (neutrophils) are rapidly recruited to the site of cell injury, providing a substantial source of reactive oxygen species that promote liver repair^20^. Subsequently, there is a massive infiltration of inflammatory monocytes, leading to monocytopenia in the circulation, reflecting the degree of liver injury^21,22^. As these monocytes differentiate into macrophages, they assume an anti-inflammatory, wound-healing phenotype, initiating the healing process and facilitating the transition from the initial inflammatory phase of acute liver injury (ALI) to the resolution phase.

Emerging therapeutic strategies have focused on modulating the immune response to enhance liver regeneration and mitigate inflammation^23,24^. Among these, macrophages, particularly alternatively activated macrophages (AAMs), have shown promise due to their roles in tissue repair and inflammation resolution^25–27^, being involved in several activities that include regulating inflammatory cells, cell killing and regulation of fibrotic processes in wound healing^28–30^. AAMs, typically induced by cytokines such as IL-4 and IL-13, are capable of phagocytosing cell debris, secreting anti-inflammatory factors, and promoting the restoration of tissue damage through the release of growth factors and angiogenic mediators^31^.

The efficacy of bone marrow (BM)-derived macrophages has been demonstrated in a murine liver fibrosis model, improving liver regeneration and function^32^, and the safety and feasibility of a peripheral infusion of ex vivo matured autologous monocyte-derived macrophages have been demonstrated in patients with cirrhosis^33^. Building upon the work by Starkey-Lewis et al. which demonstrated the efficacy of BM-derived AAMs in reducing hepatic necrosis and systemic inflammation in a mouse model of APAP-induced liver injury^34^, this study aims to translate these findings into a clinically viable product. We used allogeneic AAMs derived from ‘universal’ blood group O donor monocytes as a single injection, to eliminate the need for donor-recipient matching.

A cryopreserved product could be stored near the point of treatment and thawed on demand prior to infusion. A significant challenge in this translation is ensuring the stability, viability, and functionality of AAMs under clinical conditions, particularly through the processes of cryopreservation and thawing. Cryopreservation is essential for the clinical use of cell therapies as it enables long-term storage, preventing damage to the cells at low temperatures and ensuring the cells are readily available when needed. However, the freeze–thaw cycle can potentially alter cell morphology, surface marker expression, metabolic activity, and the ability to respond to and modulate inflammatory environments^35,36^.

This research focuses on the development and characterization of cryopreserved human AAMs. It particularly evaluates whether these cells, post-thaw, maintain their anti-inflammatory and regenerative properties.

Following successful in vitro validation, the efficacy and safety of these cryopreserved human alternatively activated macrophages (hAAMs) was assessed in vivo in a model of APAP-induced ALI. This is crucial for demonstrating that cryopreserved hAAMs can reduce liver injury, mitigate inflammatory responses, and enhance tissue regeneration effectively and safely, reflecting the therapeutic results achieved with fresh cells.

## Results

### Viability and functional integrity of cryopreserved hAAMs

**Fig. 1:**
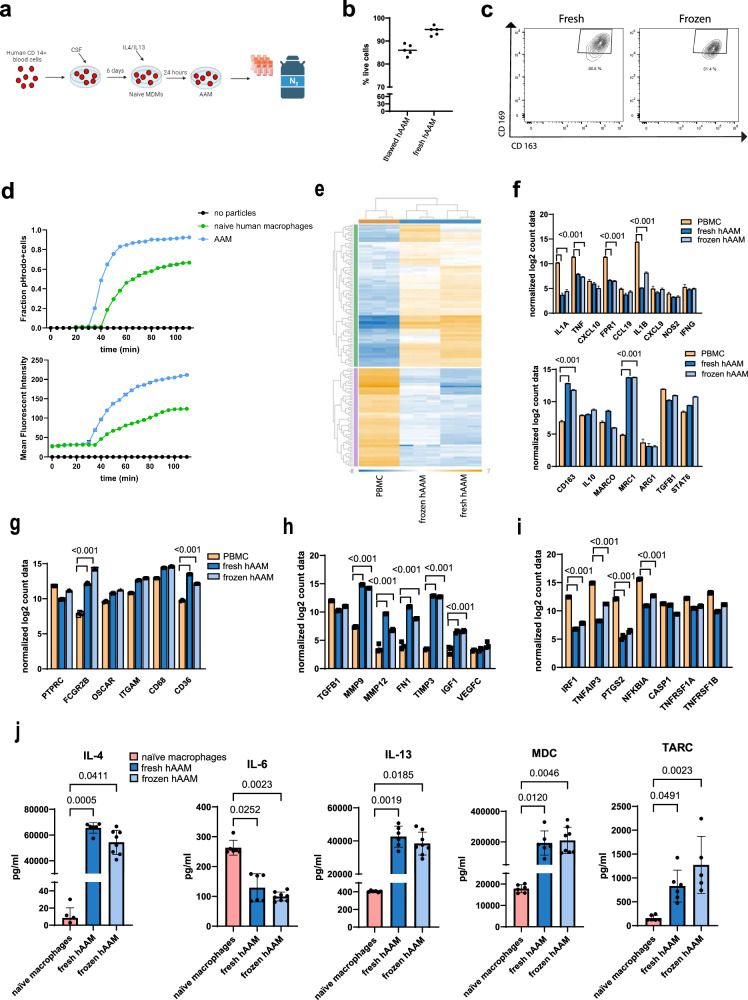
Characterization of fresh and cryopreserved hAAMs. **a** hMDMs were differentiated from CD14^+^ cells isolated from healthy volunteer buffy coats before incubation with hCSF-1 for 5 days. hAAMs were generated by stimulating hMDMs 24 h with hIL-4 and hIL-13, resuspended in Plasma-Lyte and stored at ≤−135 °C in liquid nitrogen. Created in BioRender. Candela, M. (2024) https://BioRender.com/y92e341. **b** Trypan Blue exclusion test performed on thawed and fresh hAAM. **c** Representative flow cytometry plots showing AAM macrophage markers CD169 and CD163 in fresh and thawed hAAM. **d** Phagocytosis quantification: pHrodo-positive cell fraction (up) and total cell MFI (down) conducted in human naive macrophages and hAAMs, for 180 min every 5 min. **e** Heatmap showing gene expression profiles of PBMCs, fresh hAAMs, and frozen hAAMs across a wide range of genes. The data show distinct clustering patterns for the different cell types, with fresh and frozen hAAMs sharing a similar transcriptional profile. **f** The top panel illustrates the expression level of key genes of pro-inflammatory macrophage markers in PBMCs, fresh hAAMs, and frozen hAAMs; the bottom panel illustrates the expression level of key genes of AAMs markers in PBMCs, fresh hAAMs, and frozen hAAMs. **g** Expression level of key phagocytosis-related genes in PBMCs, fresh hAAMs, and frozen hAAMs. **h** Expression of genes associated with regeneration in PBMCs, fresh hAAMs, and frozen hAAMs. **i** Expression levels of genes involved in apoptosis and cell survival in PBMCs, fresh hAAMs, and frozen hAAMs. **j** Expression of cytokines (IL-4, IL-6, IL-13, MDC, TARC) in fresh and frozen cell culture medium. Mean ± SD, *n* = 4–8; comparison was done with one-way ANOVA test or post hoc test, significant diff. among means (*P* < 0.05).

Building upon our previous studies demonstrating the potential of mouse AAMs as a cell-based therapy in APAP-ALI mice^34^, we aimed to evaluate the effectiveness of cryopreserved hAAMs in promoting necrosis resolution and mitigating the inflammatory response in an ALI model, the APAP-ALI mouse model. hAAMs were differentiated from human peripheral CD14^+^ blood mononuclear cells (PBMCs) obtained from UK blood donors, treated with CSF1 for 6 days, and the cytokines IL-4/IL-13 for 24 h (Fig. 1a). hAAMs were cryopreserved in Plasma-Lyte solution at ≤−135 °C.

To assess the effect of the cryopreservation process on hAAMs, we determined cell viability and the retention of functional markers crucial for their therapeutic efficacy. hAAM cell viability was evaluated using Trypan Blue exclusion, which demonstrated a survival rate of 87% in thawed cells and 95% in freshly isolated hAAMs (Fig. 1b). This suggests minimal cell loss due to cryopreservation and underscores the effectiveness of our cryopreservation protocol.

The functional integrity of the cryopreserved hAAMs was evaluated by analyzing the expression of key surface markers associated with their regenerative and anti-inflammatory functions. Flow cytometry was used to quantify the expression of CD169^37,38^ and CD163^39,40^, indicative markers of alternative activated macrophages. The results showed that the expression of these markers in cryopreserved hAAMs was comparable to fresh hAAMs, with no significant degradation observed post-thaw (*p* > 0.05) (Fig. 1c), indicating the preservation of their surface phenotype.

In addition to surface marker retention, the phagocytic activity of the cryopreserved hAAMs was evaluated in vitro using fluorescently pHrodo™ dye-conjugated particles (Fig. 1d). The quantification of phagocytosis was expressed as the fraction of pHrodo-positive cells and the relative mean fluorescence intensity (MFI). This assay demonstrated that hAAMs exhibited more phagocytic activity compared to human naive macrophages, as they were able to phagocytize pHrodo-bioparticles faster (after 30 min vs 40 min in the naive group). Moreover, the MFI was higher at 30 min and throughout the entire experiment in hAAMs compared to human naive macrophages, indicating the retention of their alternative activation phenotype.

To deeper characterize the molecular expression of fresh and cryopreserved hAAM, we performed a NanoString analysis. As shown in Fig. 1e, both fresh and frozen hAAMs exhibited similar gene expression profiles, distinct from PBMCs. Key surface markers associated with AAMs, such as CD163, Marco, and Stat6, were maintained post-cryopreservation (Fig. 1f, bottom panel). Importantly, markers associated with pro-inflammatory macrophages (e.g., Fpr1, Tnf-α, and IL-1β) were significantly lower in hAAMs compared to PBMCs, confirming the alternatively activated phenotype of both fresh and frozen hAAMs (Fig. 1f, top panel). Phagocytosis-related markers, including Fgcr2b and CD36, were significantly expressed in both fresh and frozen hAAMs, confirming retained phagocytic capacity after freezing (Fig. 1g). In addition, frozen hAAMs retained their ability to secrete important cytokines, including IL-10 and Tgf-β (Fig. 1f, bottom panel), and expressed higher levels of tissue-repair factors such as Mmp9, Mmp12, and Vegf, similar to fresh hAAMs (Fig. 1h). Finally, markers related to apoptosis and cell survival, such as Irf-1, Ptgs2, and Casp-1 remained consistent between fresh and frozen hAAMs, indicating that the freeze-thaw process did not affect their functional capacity (Fig. 1i).

Finally, secretome analysis (Fig. 1j) of fresh and frozen hAAMs showed similarly high levels of expression for markers of alternatively activated macrophages, such as IL-4, IL-13, MDC, and TARC, and undetectable expression of cytokines such as IL-6, TNF-α, and IL-12p70 (data not shown).

These results demonstrate that cryopreserved hAAMs retain their functional properties, including phagocytosis, suggesting that the functional activities essential for their therapeutic application remain intact post-thaw.

### Efficacy evaluation of cryopreserved hAAMs in vivo

Next, we tested whether cryopreserved hAAM treatment is non-toxic and effective in vivo. To investigate the potential therapeutic application of hAAMs, we assessed the effect of cryopreserved hAAMs in the APAP-ALI mouse model.

**Fig. 2:**
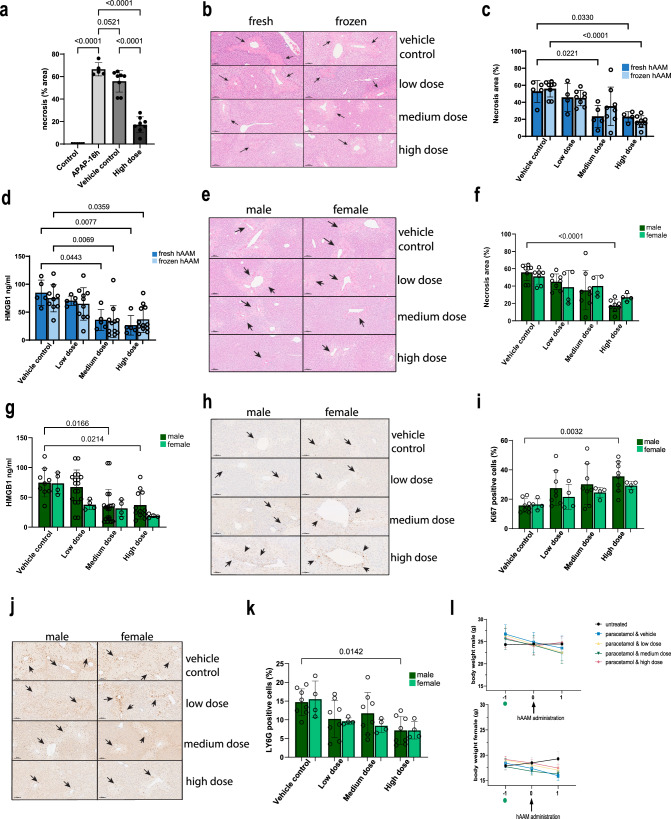
Efficacy of fresh/cryopreserved hAAM in male and female APAP mouse model. **a** Necrosis quantification of liver tissues from APAP-ALI mice, injected with vehicle control or high dose of thawed hAAM (1 × 10^6^). **b** Representative H&E liver staining from APAP-ALI mice receiving indicated treatments of fresh and frozen hAAM (low dose: 0.25 × 10^6^, medium dose 0.5 × 10^6^, and high dose 1 × 10^6^) at 16 h after APAP injury. The black arrows indicate the damaged areas. **c** Necrosis quantification of liver tissues from APAP-ALI mice, injected with vehicle control or three different doses of fresh and thawed hAAM. **d** HMGB1 analysis of serum from APAP-ALI mice treated with vehicle control and the three doses of fresh and thawed hAAM. **e** Representative H&E liver staining from APAP-ALI male and female mice receiving indicated treatments of thawed hAAM (low dose: 0.25 × 10^6^, medium dose 0.5 × 10^6^, and high dose 1 × 10^6^) at 16 h after APAP injury. The black arrows indicate the damaged areas. **f** Necrosis quantification of liver tissues from APAP-ALI male and female mice, injected with vehicle control or three different doses of thawed hAAM. **g** HMGB1 analysis of serum from APAP-ALI male and female mice treated with vehicle control and the three doses of thawed hAAM. **h** Representative Ly6G immunostaining in liver sections from APAP-ALI male and female mice receiving indicated treatments. The black arrows indicate the Ly6G-positive cells. **i** Quantification of Ly6G immunostaining of liver tissues from APAP-ALI male and female mice, injected with vehicle control or three different doses of thawed hAAM. **j** Representative Ki67 immunostaining in liver sections from APAP-ALI mice receiving indicated treatments. The black arrows indicate the Ki67-positive cells. **k** Quantification of Ki67 immunostaining of liver tissues from APAP-ALI mice, injected with vehicle control or three different doses of thawed hAAM. **l** Body weight of male (top panel) and female (bottom panel) mice measured pre- and post-thawed hAAM administration in all five groups, as shown in the legend. APAP was administrated on day −1 (green dot). Mean ± SD, *n* = 4–8; comparison was done with one-way ANOVA test or post hoc test, significant diff. among means (*P* < 0.05).

First, we assessed the efficacy of cryopreserved hAAMs in vivo, in an APAP mouse model. Sixteen hours after an APAP overdose, when necrosis was already established, hAAMs or vehicle control were injected intravenously into the mice. Another 16 h later, the mice were analyzed. First, we quantified the necrosis at 16 h post-APAP administration, to establish a baseline of liver injury, helping to demonstrate any potential differences in necrosis reduction between 16 and 36 h post-APAP in control versus treated animals (Fig. 2a).

Then, we evaluated the distribution of liver necrotic areas and the serum concentration of the translational biomarker HMGB1 in APAP-injured mice treated with both fresh and thawed hAAMs at different concentrations—low dose (0.25 × 10^6^ cells), medium dose (0.5 × 10^6^ cells), and high dose (1 × 10^6^ cells). H&E staining and relative quantification of liver sections revealed a significant decrease in necrotic tissue distribution in mice treated with the highest dose of thawed cryopreserved hAAMs (Fig. 2b, c), comparable to the fresh hAAMs. Consistent with H&E quantification, HMGB1 ELISA analysis indicated a significant reduction in serum HMGB1 (Fig. 2d) following treatment with medium and high doses of cryopreserved hAAMs. Serum analysis of alanine aminotransferase (ALT) (Supplementary Fig. 1a) and bilirubin (Supplementary Fig. 1b) did not show a significant difference between the two groups. These results indicated comparable results with fresh and cryopreserved AAMs, suggesting a dose-dependent reduction in liver necrosis, and corroborating the hypothesis that cryopreserved hAAMs retain their abilities to reduce the hepatic necrosis.

Considering the sex-dependent susceptibility to APAP-induced liver injury^41–44^, we conducted experiments in both male and female APAP-ALI mice treated with the three doses of cryopreserved hAAMs. Serum activity of ALT, aspartate aminotransferase (AST), glutamate dehydrogenase (GLDH), and bilirubin showed no change with cell treatment in males and females (Supplementary Fig. 1d–f). A decrease in necrotic areas was observed in both sexes following hAAMs administration, with statistical significance in males receiving the high dose (Fig. 2e, f). This reduction correlated with serum HMGB1 (Fig. 2g), which was decreased in both males and females. These data support the ability of hAAMs to ameliorate liver damage in both sexes. Assessment of hepatocyte proliferation using the proliferation marker Ki67 (Fig. 2h) revealed an increase in both males and females treated with medium and high doses of hAAMs, with statistically significant proliferation observed in males receiving the high dose (Fig. 2i). The expression of the neutrophil marker Ly6G (Fig. 2j) was analyzed in the liver tissue of the same APAP-ALI mice. hAAMs administration resulted in a significant reduction in Ly6G expression in both sexes, with males treated with the high dose exhibiting a statistically significant decrease (Fig. 2k).

Lastly, the body weight of male and female mice (Fig. 2l top and bottom panel respectively) was monitored throughout the study. While both cohorts showed weight loss upon APAP administration, male mice treated with the high dose of hAAMs regained body weight after hAAMs administration and female mice did not show any loss of body weight after cells injection.

Our results demonstrate that cryopreserved hAAMs retain their functional properties, and effectively reduce liver necrosis and suppress inflammation in the APAP-ALI mouse model. The comparable efficacy of fresh and thawed hAAMs supports the potential clinical application of cryopreserved hAAMs. Furthermore, the findings indicate that hAAMs treatment promotes liver proliferation, decreases necrotic tissue, and mitigates the inflammatory response in both male and female mice, highlighting the therapeutic potential of hAAMs for the treatment of APAP-induced ALI in a sex-independent manner.

### Toxicological assessment of high-dose hAAM therapy in healthy mice

To explore the safety of higher therapeutic doses of cryopreserved hAAMs ahead of potential clinical use, we conducted a toxicological assessment in healthy mice. This study was designed to detect any adverse effects from escalating doses of hAAMs.

Mice were administered one of three doses of cryopreserved hAAMs—low dose (1 × 10^6^ cells), medium dose (2 × 10^6^ cells), and high dose (3 × 10^6^ cells). Post-administration, a 24-hour monitoring period was observed to assess any immediate toxic effects.

**Fig. 3:**
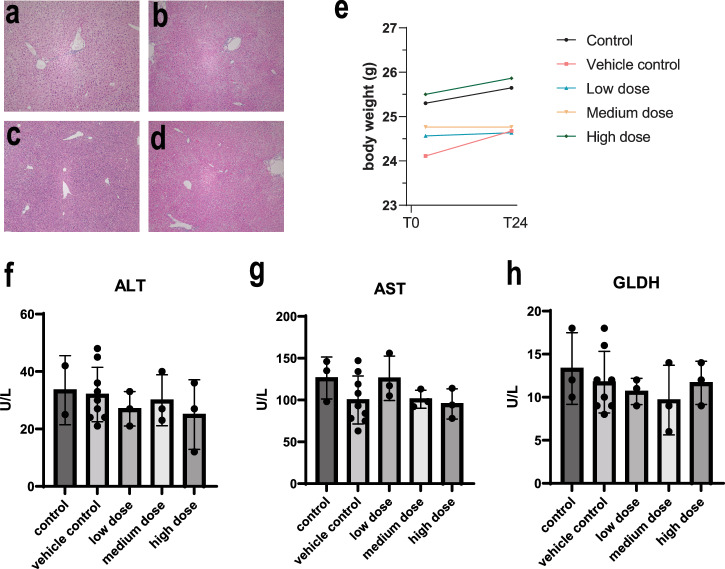
Higher doses of hAAMs do not induce any toxic effect in healthy mice. Representative H&E staining of liver sections from healthy mice injected with vehicle control (**a**) and three different doses of thawed hAAM: (**b**) low dose: 1 × 106, (**c**) medium dose: 2 × 10^6^, and (**d**) high dose: 3 × 10^6^, for 24 h. **e** Mouse body weight was measured at T0, when the cells were injected, and T24, after 24 h. **f**–**h** Serum ALT, AST, and GLDH activity measured in healthy mice receiving vehicle control and three different doses of thawed hAAM: low dose: 1 × 10^6^, medium dose: 2 × 10^6^ and (**d**) high dose: 3 × 10^6^, for 24 h. Mean ± SD, *n* = 3–8; comparison was done with one-way ANOVA test or post hoc test, significant diff. among means (*P* < 0.05).

Following the treatment period (24 h), critical organs, including the liver, lungs, heart, kidneys, and spleen were collected and subjected to histological examination using H&E staining (Fig. 3a–d) (Supplementary Fig. 2a–p). The results revealed no morphological alterations or pathological damage in any of the examined tissues, indicating the absence of gross organ toxicity at all administered doses.

We also monitored systemic effects by measuring changes in body weight (Fig. 3e) and conducting blood analyses (Fig. 3f–h) to assess liver function. Body weight measurements taken before and after cryopreserved hAAM administration showed no significant changes, reinforcing the absence of systemic toxicity. Furthermore, serum concentration of liver-specific enzymes—ALT, AST and GLDH—(Fig. 3f–h) remained within normal ranges across all treatment groups, suggesting that high-dose hAAM administration does not impair liver function in healthy mice.

The lack of histopathological changes and the stability of body weight and liver enzymes suggest that high doses of hAAMs were well-tolerated without inducing any adverse effects in healthy mice. These findings are crucial for establishing the safety of hAAMs when considering dose escalation in clinical settings, particularly for conditions like ALI where higher doses may be necessary for effective treatment.

### hAAMs improve survival and regeneration in a severe model of APAP-ALI

**Fig. 4:**
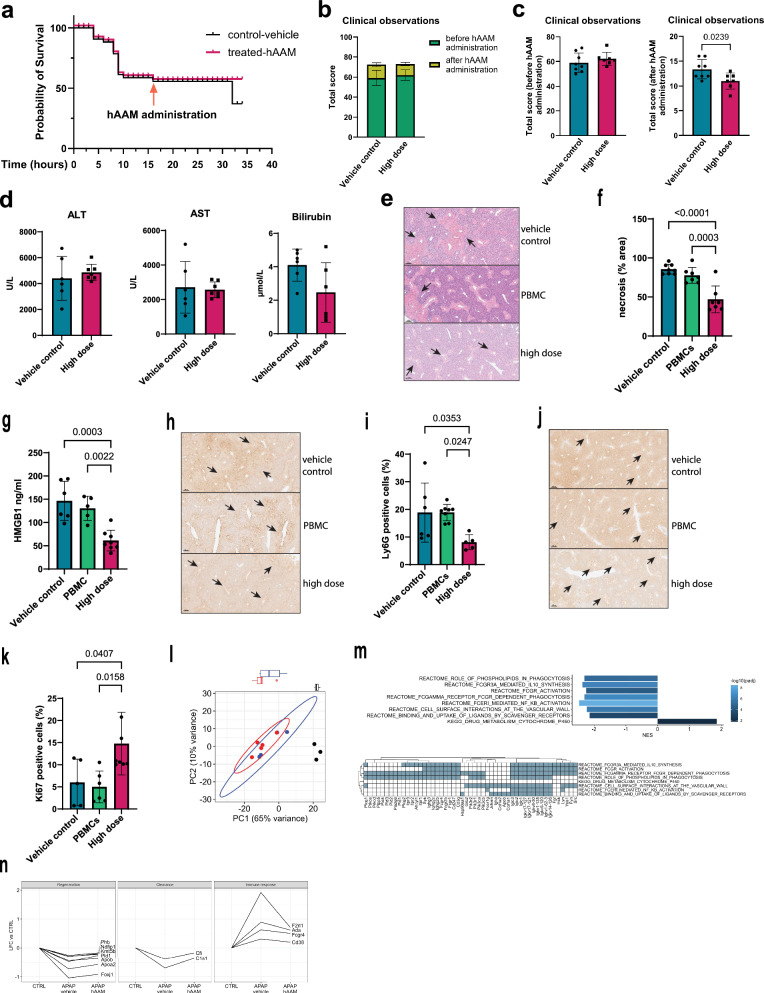
hAAMs efficacy in a severe model of APAP-ALI. **a** Survival curve (32 h) of mice subjected to 500 mg/kg APAP injury, and administrated vehicle control (black line) or high dose of hAAM (1 × 10^6^ cells) (red line) 16 h later, then culled 16 h after the treatment. **b** Total clinical observations score of mice, measured before and after hAAMs administration. Mice were assessed on five parameters (hunching, piloerection, neurological symptoms, responsiveness to touch, skin paleness, and breathing), with a score ranging from 0 to 3 (see Table 1); the total score represents the cumulative sum of all scores. APAP treatment and vehicle: vehicle control, APAP treatment, and high dose hAAMs: disease induction plus high dose hAAMs (1 × 10^6^). Mean ± SD, *n* = 7–8. **c** Total clinical observations score of mice, measured before (left panel) and after (right panel) hAAMs administration. **d** Serum ALT, AST, and bilirubin levels measured 32 h post-injection in APAP-ALI mice receiving the indicated treatments (high dose of hAAM: 1 × 10^6^ cells) and vehicle control 16 h post-APAP injury. **e** Representative H&E liver staining from APAP-ALI mice receiving indicated treatments of thawed hAAM (high dose 1 × 10^6^) and PBMCs (high dose 1 × 10^6^), and vehicle control, at 16 h after APAP injury. The black arrows indicate the damaged areas. **f** Necrosis quantification of liver tissues from APAP-ALI mice, treated with hAAM, PBMCs, and vehicle control. **g** HMGB1 analysis of serum from APAP-ALI mice treated with hAAM, PBMCs, and vehicle control. **h** Representative Ly6G immunostaining in liver sections from APAP-ALI mice receiving indicated treatments. The black arrows indicate the Ly6G-positive cells. **i** Quantification of Ly6G immunostaining of liver tissues from APAP-ALI mice, treated with hAAM, PBMCs, and vehicle control. **j** Representative Ki67 immunostaining in liver sections from APAP-ALI mice receiving indicated treatments. The black arrows indicate the Ki67-positive cells. **k** Quantification of Ki67 immunostaining of liver tissues from APAP-ALI mice treated with hAAM, PBMCs, and vehicle control. Mean ± SD, *n* = 7–8. Comparison was done with one-way ANOVA test or post hoc test, significant diff. among means (*P* < 0.05). **l** Principal component analysis of all RNA-seq samples, based on the 500 most variable genes. Ellipses represent 95% confidence for groups with ≥4 samples. Boxplots show the distribution of samples along PC1, split by treatment group. Black dot: CTRL, red dot: APAP-vehicle control, blue dot: APAP-hAAM. **m** Top panel: bar plot of selected Reactome and KEGG gene sets significantly enriched between APAP-Vehicle control and APAP-hAAM conditions. Normalized enriched score (NES) is plotted for each gene set; bars are colored by significance, adjusted for multiple comparisons. Bottom panel: Matrix of genes associated with ≥2 gene sets identified above. **n** Plots of log2 fold change (LFC) in APAP-Vehicle control and APAP-hAAM group mice compared to CTRL, for selected genes associated with regeneration, clearance, and immune response. Lines connect results for each gene. All genes have differential expression significant (FDR < 0.05) between CTRL and APAP-Vehicle control, but non-significant between CTRL and APAP-hAAM.

To further assess the therapeutic efficacy of hAAMs in a more severe model of liver injury, we administered a 500 mg/kg dose of APAP to induce ALF. hAAM treatment improved survival outcomes compared to vehicle controls, as shown in Fig. 4a, with hAAM-treated mice demonstrating greater survival rates post-APAP administration. Standard clinical features were scored using an established phenotypic system (Supplementary Table 1)^34^. Mice were assessed every hour (Fig. 4b) for the following patterns: hunching, piloerection, neurological symptoms, responsiveness to touch, skin paleness, and breathing, previous (Fig. 4c left panel) and after (Fig. 4c right panel) hAAMs administration, and the higher score indicates poorer health. hAAM-treated animals exhibited fewer clinical signs of distress, such as decreased mobility and lethargy, as shown by the clinical observations in Fig. 4c (right panel), with a notable improvement in overall activity levels.

Liver injury markers, including ALT, AST, and bilirubin, were measured to assess liver function (Fig. 4d). While the high dose of APAP resulted in elevated levels of ALT, AST, and bilirubin, hAAM treatment attenuated these elevations, although not reaching statistical significance. A significant decrease in necrotic areas was observed in mice following hAAM administration, compared to mice receiving either vehicle control or frozen Peripheral Blood Mononuclear Cells (PBMC) (Fig. 4e, f). This reduction correlated with serum HMGB1 (Fig. 4g). Administration of hAAMs resulted in a significant reduction in Ly6G expression (Fig. 4h, i) and an increase in the proliferation marker Ki67 (Fig. 4j, k), suggesting that hAAMs effectively reduced necrotic cell death and promoted liver regeneration, even in the context of severe liver injury.

These results demonstrate that hAAMs can mitigate the effects of high-dose APAP-induced liver injury, supporting their potential as a therapeutic intervention in more extreme cases where liver regeneration is impaired.

### hAAM-mediated modulation of liver regeneration and immune suppression in APAP-induced liver injury

To investigate the mechanisms by which hAAMs promote liver protection and recovery, we conducted bulk RNA sequencing on control mice, APAP-injured mice treated with vehicle, and APAP-injured mice treated with hAAMs. Differential expression analysis showed no statistically significant differences between the vehicle-treated and hAAM-treated groups. However, principal component analysis (PCA) (Fig. 4l) revealed that the gene expression profile of hAAM-treated mice was closer to the healthy control group along PC1 (associated with APAP injury) compared to the vehicle-treated mice.

Gene set enrichment analysis (Fig. 4m) identified pathways significantly enriched in the APAP-hAAM group compared to the APAP-vehicle control group, including pathways associated with phagocytosis, IL-10 synthesis, NF-κB signaling, and scavenger receptor activity—key processes involved in the activity of alternatively activated macrophages.

Notably, genes related to regeneration (e.g., Phb, Ndfip1, and Foxj1), and clearance (e.g., Cfi and C1s1), had significantly lower expression in the vehicle-treated group compared to the healthy control group; expression of these genes was higher in hAAM-treated mice, with no significance difference in expression compared to control (Fig. 4n). Conversely, genes associated with the immune response (e.g. Cd38, F2rl1, and Ada) were significantly upregulated in the vehicle-treatment group compared to control, and reduced in the hAAM-treated group to a level not significantly different to control.

These findings suggest that hAAMs may support liver regeneration and modulate immune responses, contributing to liver recovery. However, further analysis is needed to fully elucidate the specific mechanisms by which hAAMs exert these protective effects.

### Biodistribution assessment confirms rapid clearance of hAAMs from liver, lung, and spleen within 2 weeks after injection

**Fig. 5:**
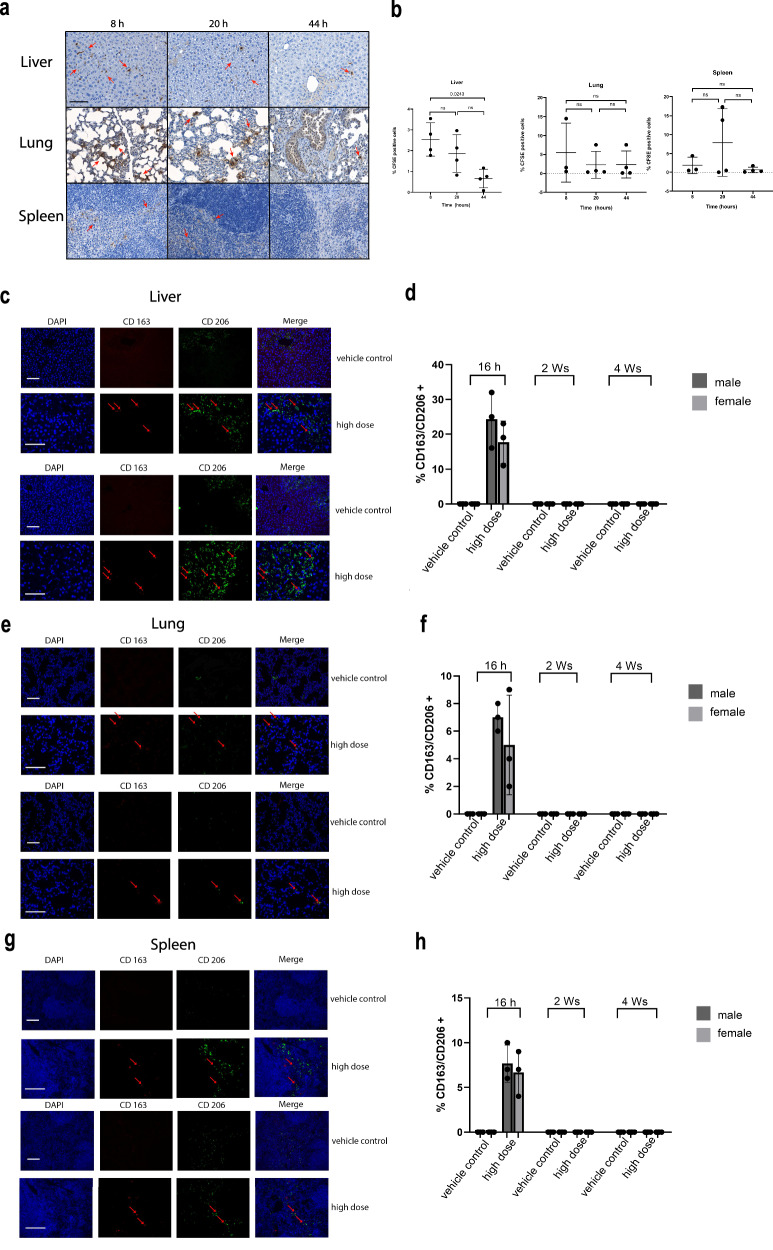
Rapid clearance of hAAMs from liver, lung, and spleen within two weeks post-administration. **a** Representative staining of liver, lung, and spleen tissues for CFSE-labeled cells of APAP-ALI mice at 8, 20, and 44 h after 1 × 10^6^ mAAMs administration. **b** Quantification of % CFSE positively stained nuclei in the liver, lung, and spleen tissues of APAP-ALI mice at 8, 20, and 44 h after 1 × 10^6^ mAAMs administration. Mean ± SD, *n* = 3–4; one-Way-ANOVA with Brown-Forsythe and Welch multiple comparisons correction was performed to analyze for changes across time points within the same organ. **c** Representative double staining of CD163 (red), CD206 (green), and nuclei (blue) in liver tissue of APAP-ALI male (top panel) and female (bottom panel) mice at 16 h after vehicle control or hAAMs (high dose: 1 × 10^6^) administration. Scale bars—100 μm. **d** Quantification of double-positive cells in the liver tissue for CD163 and CD206 at 16 h, 2 weeks, and 4 weeks after vehicle control or hAAMs (high dose: 1 × 10^6^) administration in male and female APAP-ALI mice. **e** Representative double staining of CD163 (red), CD206 (green), and nuclei (blue) in lung tissue of APAP-ALI male (top panel) and female mice (bottom panel) at 16 h after vehicle control or hAAMs (high dose: 1 × 10^6^) administration. Scale bars—100 μm. **f** Quantification of double-positive cells in the lung tissue for CD163 and CD206 at 16 h, 2 weeks, and 4 weeks after vehicle control or hAAMs (high dose: 1 × 10^6^) administration in male and female APAP-ALI mice. **g** Representative double staining of CD163 (red), CD206 (green), and nuclei (blue) in spleen tissue of APAP-ALI male (top panel) and female mice (bottom panel) at 16 h after vehicle control or hAAMs (high dose: 1 × 10^6^) administration. Scale bars—100 μm. **h** Quantification of double positive cells in the spleen tissue for CD163 and CD206 at 16 h, 2 weeks and 4 weeks after vehicle control or hAAMs (high dose: 1 × 10^6^) administration in male and female APAP-ALI mice. Mean ± SD, *n* = 3; comparison was done with one-way ANOVA test or post hoc test, significant diff. among means (*P* < 0.05).

Biodistribution analysis of hAAMs was conducted to elucidate their distribution, persistence, and clearance in vivo from target and non-target tissues at multiple time points on mice receiving APAP injury. Published data demonstrate that hAAMs are unlikely to persist long-term due to immune-mediated clearance in the immunocompetent APAP-ALI mouse model^34^. Published data demonstrates the long-term persistence of this allogeneic product is unlikely due to immune-mediated clearance in the immunocompetent APAP-ALI mouse model (Starkey-Lewis et al.^34^). The time-course analysis of 1 × 10^6^ CFSE-labeled mAAMs administered to mice 16 h after APAP treatment revealed the presence of these cells in the liver at 8 h, followed by a significant decrease at 20 and 44 h. A similar trend was observed in the lungs and spleen (Fig. 5a, b) To investigate this further, we assessed the expression of hAAM markers, CD163 and CD206, at 16 h, 2 weeks, and 4 weeks after hAAMs administration in both male and female mice. Results showed the presence of hAAMs in the liver (Fig. 5c, d), lung (Fig. 5e, f), and spleen (Fig. 5g, h) tissues at 16 h after administration in both sexes. Notably, the absence of hAAMs in the heart (Supplementary Fig. 3e), intestine (Supplementary Fig. 3f), and kidney (Supplementary Fig. 3g) further contribute to the safety profile of these cells. The lack of detectable hAAMs in these vital organs indicates that hAAM administration does not cause prolonged accumulation in non-target tissues. The biodistribution analysis confirms the transient hepatic localization of hAAMs and their clearance from the liver (Supplementary Fig. 3b), lung (Supplementary Fig. 3c), and spleen (Supplementary Fig. 3d) within 2 weeks in the APAP-ALI mouse model.

The rapid clearance of hAAMs, along with their absence in the heart and intestine, supports the safety profile of these cells in treating APAP-ALI.

### Longer-term safety and therapeutic efficacy of hAAMs in APAP-ALI treatment

To further assess the safety and therapeutic potential of cryopreserved hAAMs in treating APAP-ALI, we conducted a 28-day study in both male and female mice. This investigation aimed to observe any longer-term adverse effects associated with different doses of hAAMs, as well as their effect on recovery after treatment.

Mice were treated with APAP and after 16 h were administered one of two doses of thawed hAAMs (low dose: 1 × 10^6^ and high dose: 3 × 10^6^ cells). Body weights were monitored daily for the first five days and then weekly, with a final assessment at necropsy (28 days). Despite initial weight loss due to APAP administration, both male and female mice demonstrated significant weight gain following hAAM treatment, with those receiving higher doses showing the most rapid recovery (Supplementary Fig. 4a left and right panel respectively).

Organ weights—including liver, spleen, kidney, colon, brain, heart, lung, femur, and reproductive organs—were measured at the study’s conclusion (Supplementary Fig. 5b). There were no significant changes in organ weights between the treated and control groups.

Standard clinical features were scored using the established phenotypic system (Supplementary Table 1)^34^. Mice treated with hAAMs exhibited markedly improved health scores compared to those in the control group, particularly at higher doses, suggesting an alleviation of liver injury-associated symptoms such as piloerection, hunching, breathing, and neurological signs (Supplementary Fig. 4b).

**Fig. 6:**
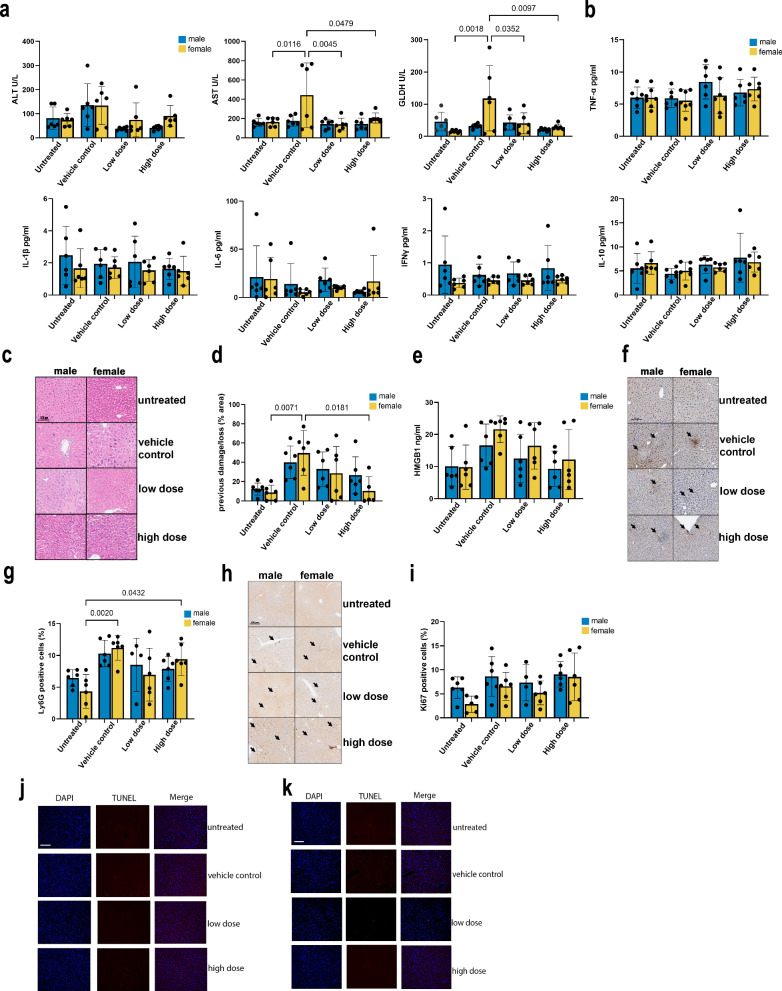
Efficacy and safety of cryopreserved hAAMs administration after 28 days in APAP-ALI mice. **a** Serum ALT, AST, and GLDH activity measured after 28 days of the injection in APAP-ALI male and female mice receiving indicated treatments (hAAM low dose: 0.25 × 10^6^ and high dose 1 × 10^6^) and vehicle control at 16 h after APAP injury. **b** Serum concentrations of pro-inflammatory cytokines measured in APAP-ALI male and female mice receiving indicated treatments. **c** Representative H&E liver staining from APAP-ALI male and female mice receiving indicated treatments of thawed hAAM (low and high dose) and vehicle control. The black arrows indicate the damaged areas. **d** Necrosis quantification of liver tissues from APAP-ALI male and female mice, injected with vehicle control or two different doses (low and high) of thawed hAAM at 16 h after APAP injury. **e** HMGB1 analysis of serum from APAP-ALI male and female mice treated, after 16 h APAP injury, with vehicle control and the two doses (low and high) of thawed hAAM. **f** Representative Ly6G immunostaining in liver sections from APAP-ALI male and female mice receiving indicated treatments. The black arrows indicate the Ly6G-positive cells. **g** Quantification of Ly6G immunostaining of liver tissues from APAP-ALI male and female mice, injected with vehicle control or two different doses of hAAM (low and high) at 16 h of APAP injury. **h** Representative Ki67 immunostaining in liver sections from APAP-ALI male and female mice receiving indicated treatments. The black arrows indicate the Ki67-positive cells. **i** Quantification of Ki67 immunostaining of liver tissues from APAP-ALI male and female mice, injected with vehicle control or two different doses (low and high) of thawed hAAM. **j**, **k** Representation of Tunel staining of liver tissues from APAP-ALI male (**j**) and female mice (**k**), receiving indicated treatments. Mean ± SD, *n* = 6; comparison was done with one-way ANOVA test or post hoc test, significant diff. among means (*P* < 0.05).

Blood analyses conducted at the end of the treatment period (28 days) revealed no significant alterations in hematocrit, hemoglobin levels, erythrocyte and platelet counts, mean cell volume, or hemoglobin concentration and levels, confirming the non-toxic nature of hAAMs even at the highest administered doses (Supplementary Fig. 4c). Lymphocyte and monocyte counts showed some variation after APAP and hAAMs administration, but without any significant impact. Liver function tests further supported these findings. The induction of liver damage leads to an increase in ALT, AST, and GLDH activity. Liver damage markers decreased upon hAAM administration, with significant results observed in AST and GLDH levels in female mice (Fig. 6a). Cholesterol and triglyceride levels appeared to decrease in both sexes in response to hAAMs administration (Supplementary Fig. 4d). The other measured parameters did not show any significant differences in either cohort.

Lastly, to evaluate the effect on inflammation after hAAMs treatment, a panel of cytokines were quantified in serum samples from APAP-ALI mice. The V-Plex analysis did not show any significant differences in the expression of TNF-α, IL-1β, IL-6, IFN-γ, IL-10 (Fig. 6b), as well IL-2, IL-5 and KG/CRO (Supplementary Fig. 5a) in either cohort.

Our findings over a 28-day period demonstrate that hAAM administration did not lead to any significant adverse effects in terms of body weight, organ pathology, blood parameters, or clinical signs. Furthermore, hAAM treatment effectively reduced markers of liver damage and modulated inflammatory responses, supporting its safety and efficacy in long-term therapeutic applications for APAP-ALI.

We also aimed to assess liver damage repair, inflammation reduction, cell proliferation capabilities, and apoptosis level in the long-term study of hAAMs where 16 hours after an APAP overdose, hAAMs were injected into the mice, and 28 days later, the mice were analyzed.

Liver tissues from male and female mice were stained with H&E to visualize previous damage/loss areas due to APAP injury (Fig. 6c). The relative quantification of these areas (Fig. 6d) was compared between mice treated with hAAMs and those treated only with APAP to evaluate the hAAMs’ effectiveness in resolving liver damage. H&E staining of liver tissues showed the presence of previous damage/loss areas resulting from APAP injury (Fig. 6c). The relative quantification revealed a significant decrease in these areas in mice treated with hAAMs after APAP-ALI disease induction, especially in females receiving higher doses hAAMs. These findings were consistent with HMGB1 results (Fig. 6e), showing a decrease in serum from both male and female mice respectively, treated with the highest hAAMs dose. This confirms the ability of hAAMs to resolve liver damage following APAP-ALI.

Immunostaining for the neutrophil marker Ly6G and its relative quantification (Fig. 6f, g respectively) were assessed to examine the impact of hAAMs on neutrophil infiltration and inflammation. The immunostaining demonstrated a significant increase in Ly6G expression in both males and females following APAP-ALI induction. However, hAAMs administration effectively reduced the expression of this marker in both sexes, with a significant decrease in female mice injected with the highest hAAMs concentration, indicating a decrease in neutrophil infiltration and inflammation caused by APAP-induced ALI.

The expression of Ki67 and its relative quantification (Fig. 6h, I, respectively) were examined to investigate hAAMs’ effect on liver cell proliferation. The results suggested an increase in liver cell proliferation in the female group after 4 weeks of liver injury induction (highest dose), without a significant difference.

Additionally, to ensure the safety of hAAM treatment, TUNEL staining was performed to detect apoptosis in liver tissues of males and females cohort (Fig. 6j, k, respectively and Supplementary Fig. 6a). The results confirmed that hAAM administration does not induce apoptosis, supporting the treatment’s safety over the extended period.

The results of our study demonstrate that hAAM administration effectively promotes liver cell proliferation and reduces liver damage and neutrophil infiltration without inducing apoptosis after 28 days in both male and female mice with APAP-ALI. The absence of any adverse effects over an extended period further underscores the possibility of using hAAM-based therapies in clinical settings.

## Discussion

There is an urgent need for innovative treatments for patients who present late with APAP-induced ALF and new strategies for ALF treatment and recovery. Recent investigations have revealed, during APAP-ALI injury, a massive infiltration of monocytes-derived macrophages (MoMFs), which differentiate into inflammatory macrophages with poor phagocytic ability^45^. In this context, the use of already polarized macrophages^46^, and in particular AAMs, known for their anti-inflammatory and tissue-repair functions, represents a potential therapeutic avenue. AAM therapy has already been tested in pre-clinical mouse models for conditions such as renal fibrosis^47^, colitis^48^, and liver fibrosis^32^, but has not yet been assessed in the setting of ALI.

This study introduces an innovative and promising cell-based immunotherapeutic approach, significantly advancing previous research^34^ by demonstrating the efficacy of cryopreserved hAAMs in APAP-ALI model. While previous studies, including Starkey-Lewis et al. work, demonstrated the therapeutic potential of mAAMs in liver injury, this study uses cryopreserved hAAMs, representing a critical advancement toward clinical relevance, addressing the challenges related to human application.

Our study highlights the therapeutic potential of cryopreserved hAAMs, which remain functionally active post-thaw, including phagocytosis and the ability to release anti-inflammatory cytokines, making them a practical option for clinical use. The ability to cryopreserve and store hAAMs ensures they can be readily available for immediate use in cases of ALF. Hepatocyte necrosis plays a pivotal role in APAP-ALI, initiating the activation of the innate immune system through DAMPs such as HMGB1. Our study demonstrates that cryopreserved hAAMs treatment leads to a specific reduction in liver necrotic areas in mice treated with hAAMs compared to those treated only with APAP, in accordance with a decrease of HMGB1 expression. Thus cryopreserved hAAMs not only alleviate necrotic liver damage but also modulate the inflammatory response with comparable efficacy to fresh hAAMs. This finding confirms that cryopreservation does not compromise the therapeutic potential of hAAMs. In particular, cryopreserved hAAMs attenuated inflammation and neutrophil infiltration in both male and female mice, a hallmark of APAP-induced ALI, contributing to the exacerbation of liver injury through the release of pro-inflammatory mediators^49^. AAMs, are known to secrete anti-inflammatory cytokines such as IL-10 and TGF-β^50^. These cytokines can modulate the activity of various immune cells in the liver, leading to reduced secretion of pro-inflammatory cytokines and chemokines, thus dampening inflammation^51,52^. The clearing of necrotic areas is essential for resolving inflammation and initiating tissue-repair processes^53^, and hAAMs contribute to this process through their phagocytic ability.

Interestingly, cryopreserved hAAMs were effective even when administered after the therapeutic window for NAC, the standard treatment for APAP overdose. This significantly expands their potential clinical application to patients who present beyond the window of NAC efficacy, a point not previously explored.

An important feature of liver regeneration after liver injury is hepatocyte and endothelial proliferation^54^. Our findings indicated that the administration of cryopreserved hAAMs induces liver regeneration, enhancing liver cell proliferation, especially when administered at higher doses in females. This observation suggested that hAAMs might have a regenerative effect on liver tissue, facilitating the restoration of hepatocyte function and contributing to liver recovery. Moreover, the absence of apoptosis, as evidenced by TUNEL staining, indicated hAAM administration does not induce apoptosis, supporting the treatment’s safety over the extended period. hAAMs contribute to a regenerative environment by modulating the local immune response or by secreting factors that directly promote hepatocyte proliferation and survival.

This study highlights the dual role of hAAMs: while they participate in phagocytosis, their most significant contribution lies in regenerative signaling, creating a local environment that supports liver regeneration and recovery. The therapeutic effect of the injected cryopreserved hAAMs is likely mediated by a combination of direct phagocytic activity and the secretion of regenerative mediators. Although the number of injected macrophages (1 × 10^6^ cells) may appear small compared to the necrotic hepatocytes present during APAP-induced liver injury, the injected macrophages modulate the local immune environment, secreting anti-inflammatory cytokines such as IL-10 and TGF-β, which reduce inflammation and promote tissue healing, and growth factors such as VEGF and HGF, which are critical for hepatocyte proliferation and the restoration of liver architecture.

Moreover, a comprehensive safety evaluation of cryopreserved hAAMs in vivo demonstrated no significant toxic effects in either male or female mice, with no observable changes in organ weight, blood parameters, or clinical signs up to 28 days post-administration. This study further confirmed the safety of hAAMs through biodistribution analysis. The observed transient hepatic localization of hAAMs following APAP-ALI induction confirmed their ability to reach the target tissue. Following an efficient clearance from the liver, lung, and spleen tissues within two weeks, there was no prolonged persistence in non-target organs. These results underscore the suitability of cryopreserved hAAMs as a safe and scalable cell therapy for ALI.

In summary, the ability of cryopreserved hAAMs to mitigate liver damage and promote regeneration suggests that they could be a viable therapeutic option for APAP-induced liver injury. By demonstrating that cryopreserved hAAMs retain their efficacy post-thaw and are effective even beyond the therapeutic window for NAC, this study highlights their potential as an off-the-shelf therapeutic option. This study advances the field of liver injury treatment by demonstrating the efficacy of cryopreserved hAAMs in reducing liver damage, modulating inflammation, and promoting liver regeneration without inducing apoptosis. Based upon these results, a phase 1 clinical trial is now recruiting to determine the safety and tolerability of cryopreserved hAAMs in patients with APAP-induced hepatotoxicity (ISRCTN number: ISRCTN12637839).

## Methods

### Animals

Ten- to twelve-week-old C57BL/6 J mice, averaging 25–30 g for males and 16–20 g for females, were purchased from Charles River Laboratories (UK). All animals were housed in an environmentally controlled room with a 12-h light/dark cycle and had free access to food and water. The mice were anaesthetized with isoflurane for cardiac exsanguination to collect blood, then culled via cervical dislocation; death was confirmed by exsanguination through severing the brachial plexus. All animal experiments were conducted in accordance with the criteria outlined in a license granted under the Animals (Scientific Procedures) Act 1986 and were approved by the University of Edinburgh Animal Ethics Committee.

### Experimental design

Ten/twelve-weeks-old male or female C57BL6/JOlaHsd mice were intraperitoneally (i.p.) injected with 350 or 500 mg/kg paracetamol (Sigma Aldrich) dissolved in warm sterile saline (PanReac Applichem) or saline vehicle after 12 h fasting. Standard chow and wet mash were returned to mice 20 min post-paracetamol administration. These mice were monitored frequently (every 3 h in the first 14 h after APAP administration or every 1 h when 500 mg/Kg was used, every 8 h on the first day and daily onwards until the end of the study. Thawed PBMC, macrophages (fresh or thawed hAAMs, or mAAMs) or vehicle control (phosphate-buffered saline (PBS) ± CryoStor cell cryopreservation media (CS10 Medium), 66% CS10 medium in 1× PBS) were diluted in PBS ± 66% CS10 to reach the required final concentration for each treated arm. 100 µl of cells at the desired concentration, were intravenous (i.v.) injected via tail vein at 16 h after APAP injury, and then the animals were transferred to a warming cabinet (28 °C). After 36 h and 28 days APAP treatment, mice were humanely culled, and whole blood samples were collected via cardiac puncture into tubes ± ethylenediaminetetraacetic acid (EDTA) for hematology and blood chemistry study. A part for the liver and the lung previously perfused with 10 ml of PBS, spleen, kidney, heart, brain, femur, testis, seminal vesicles, ovary, and uterus were removed, washed, weighed, and fixed in 10% of formalin for 8 h.

### hMDMs and hAAMs production

CD14^+^ monocytes are isolated from a UK donor leukapheresis product using the CliniMACS Prodigy® closed system. CD14^+^ monocytes are cultured for 6 days (37 °C, 5% CO_2_) in low adhesion culture bags (Miltenyi) in the presence of TexMACS^TM^ serum-free media (Miltenyi) and 24,600 IU/ml CSF1 (100 ng/ml, R&D Systems), with an additional feed at day 1 to generate hMDMs. To polarize hMDM in hAAM, additional cytokines IL-4 and IL-13 were added on day six of culture at 400 IU/ml and 60 IU/ml, respectively. At day seven cells are harvested and washed using PBS/EDTA buffer and resuspended in Plasma-Lyte. They are then formulated at the required final cell density in cryopreservation media and filled into CryoMACS bags and vials. Cells are cryopreserved using a controlled rate freezer and stored in vapor phase LN2. Successful hMDM differentiation was confirmed using flow cytometry to demonstrate a ≥5-folds increase in MFI on CD206 and 25F9 expression compared to initial CD14^+^ cells from day 0. Cryopreserved hAAMs stored at ≤−135 °C in liquid nitrogen were incubated in a water bath for 5 min at 37 °C to thaw. Cell viability was performed using Trypan Blue staining and Burker Haemocytometer Counting Chambers before the injection, and the cells were maintained at 4 °C until the time of injection (~10 min).

### BMDM isolation and purification

BMDMs were prepared as previously described^32^. Mouse BM was flushed from femurs and tibias of healthy C57BL/6JOlaHsd male mice (8–10 weeks old, Charles River Laboratories). BM suspensions were filtered (70 µm), and incubated into ultra-low attachment flasks (Corning Inc.) in the presence of DMEM:F12 (1:1) cell culture media (Gibco) supplemented with FBS (10%), L-glutamine (2 mM), penicillin/streptomycin (100 U/ml, 100 µg/ ml) and murine recombinant CSF1 (40 ng/ml; Peprotech), for 6 days (37 °C, 5% CO2), with additional feeds on day 3 and 5 (100 ng/ml CSF1, in a 50% media change). mAAMs were polarized with interleukin IL-4 and IL-13 (20 ng/ml each, Peprotech) overnight. mAAMs were labeled with CellTrace CFSE (ThermoFisher), following the manufacturer’s instructions.

### NanoString nCounter analysis system

RNA was harvested from cells using the RNeasy Mini Kit (Qiagen) according to the manufacturer’s instructions. RNA was quantified using Nanodrop ND-1000 (ThermoScientific). All the samples were analyzed following the manufacturer’s protocol for a set of genes (NanoString Human Myeloid Innate Immunity Panel) using the NanoString nCounter Analysis System. Briefly, total RNA was mixed with hybridization buffer and hybridized at 65 °C overnight. Sample, wash reagents, and imaging cartridge were then processed on the nCounter Prep Station and finally imaged on the nCounter Digital Analyzer. Data generated by the nCounter system have to be normalized prior to being used to quantify gene expression and compare expression rates between different experimental conditions. Data were analyzed with nSolver 4.0™ Data Analysis and Rosalind Data Analysis.

### RNA extraction for sequencing

For bulk RNA sequencing on liver tissue from APAP-treated mice, ~10 mg frozen liver tissue was homogenized in 900 µL Qiazol lysis reagent (Qiagen) using the tissue tearor. Chloroform was mixed and incubated (5 min) before centrifugation (12,000 × *g*, 15 min, 4 °C). Aqueous phase was separated, and RNA was extracted using the RNeasy Mini Kit (Qiagen) according to the manufacturer’s instructions.

### RNA-seq data preprocessing

Read quality was initially assessed using FastQC (v.0.12.1); quality reports were collated with MultiQC (v.1.25). Illumina standard adapter sequences were trimmed using Cutadapt (v.4.6^55^); low-quality bases (Phred<20) were also trimmed. A second round of quality assessment with FastQC confirmed adapter contamination of <0.1% per sample in all samples. Paired-end sequence reads were aligned to the mouse reference genome (GRCm38.100) with STAR using the default settings (v.2.7.1a^56^). Data was imported into R, and reads were counted using the summarizeOverlaps function from the GenomicAlignments Bioconductor package (v.1.34.1^57^). Genes were filtered using the filterByExpr function in the edgeR Bioconductor package (v.3.40.2^58^), using the default parameter settings and grouping samples by treatment group.

### RNA-seq data analysis

Differential expression was computed using DESeq2 (v.1.38.3^59^). Genes with FDR < 0.05 were judged to be significant. Gene set enrichment analysis was computed for the RNA-seq and proteomics datasets using the fgsea Bioconductor package (v.1.24.0^60^) with the MSigDB Hallmark^61^, C2 (curated gene sets) and C5 (Gene Ontology subcollection; biological process component) gene sets applied to differential expression results. Gene sets with fewer than 15 genes or more than 500 genes were excluded from analysis; the number of permutations was set to 10,000. Gene sets with FDR < 0.05 were judged to be significant. For visualization of RNA-seq data, read counts were normalized with respect to library size using the regularized log (rlog) transform^59^.

The RNA-seq data associated with this study has been deposited in NCBI Gene Expression Omnibus (accession number GSE280475).

### Phagocytosis assay

naive hMDMs and hAAM were differentiated as described above. Cells were plated in 96-well CellCarrier microplates (PerkinElmer) overnight according to the manufacturer’s instructions. Macrophages were stained with NucBlue live cell stain (ThermoFisher) and CellMask Deep Red plasma membrane stain (ThermoFisher) according to the manufacturer’s instructions and transferred to Operetta high-content imaging system (PerkinElmer). Phagocytosis was initiated by the addition of pHrodo™ BioParticles™ Conjugates (ThermoFisher). Fluorescent images were taken at Opera Phenix™ in the 4′,6-diamidino-2-phenylindole (DAPI) channel, 488 nm, and 647 nm before, and at 5 min intervals after the addition of bioparticles for a maximum of 150 min. Images were quantified on Columbus image analysis software (PerkinElmer). Macrophages positive for phagocytosis were classified based on a fluorescence intensity (488 nm) greater than 500 and expressed as a fraction of all live cells (NucBlue positive cells). Mean fraction values were taken from four separate wells per group.

### Flow cytometry for human macrophages

Cells were counted and stained using a panel of antibodies to target cell surface markers with appropriate controls (i.e. unstained, fluorescence minus one). Non-specific antibody binding was blocked by incubating cells with 10% BSA for 15 min at 4 °C followed by incubation with combinations of primary antibodies (1:200 dilution) for 30 min at 4 °C in the dark. The following conjugated antibodies were used: DAPI (BioLegend), CD45 VioBlue (Miltenyi Biotec), CD14 FITC (BioLegend), CD86 PE (BioLegend), CD192 PE (BioLegend), CD169 APC (BioLegend), CD163 APC/FIRE (BioLegend). Appropriate compensation controls were used to negate spectral overlap. Samples were analyzed using the BD LSR Fortessa 5 laser (BD Biosciences, USA).

### Hematoxylin and Eosin staining and necrosis quantification

Four-micron sections of formalin-fixed paraffin-embedded (FFPE) block slides were dewaxed, rehydrated, stained with H&E, and mounted with ClearVue mountant (ThermoScientific). For necrosis quantification, H&E sections were scanned on a Vectra Polaris Automated Quantitative Imaging System (PerkinElmer) using multispectral imaging. At least six/seven 10x ROIs (representing a minimum combined tissue area of 15.5 mm^2^) were analyzed for each sample with InForm automated image analysis software (AKOYA Biosciences), which allows to design an algorithm based on the presence of the healthy, necrotic, and previous damaged tissue. After training using a representative image from every sample, and then batch processed on all images, necrosis was quantified by expressing necrotic tissue area as a percentage of total tissue area.

### Immunodetection assays and quantification

Four-micron sections of FFPE) blocks slides were dewaxed and rehydrated before heat-induced antigen retrieval. After blocking in protein solution (Spring Bioscience) for 30 min, the slides were incubated overnight with primary antibodies at 4 °C: anti-mannose receptor Abcam, Anti-CD163 Biorad, Anti-Ly6G Biolegend, Anti-Ki67 Abcam, FITC 71–900 (Invitrogen) or isotype-control IgG (Vector Laboratories). For immunofluorescence, the slides were incubated with secondary antibodies for 1 h in the dark: Alexa Fluor® 488, 647 conjugate, and DAPI (SIGMA ALDRICH, 1 µg/ml, 10 min) was applied before mounting in Fluoromount-G (Invitrogen). The intensity of the fluorescent signal is quantified in DAPI-positive nuclei. For quantification, six/seven fields per tissue were analyzed at Polaris multispectral analysis (PerkinElmer) and InForm (AKOYA Biosciences) automated image analysis software. After segmentation of the tissues and the scoring of all the images based on the positivity threshold, an algorithm created on the presence of the double positive cells was designed, expressing the percentage of the total cells number. For immunohistochemical staining, a biotinylated secondary antibody was incubated (1:500) for 1 h before treatment with an avidin-based peroxidase reagent (Vector Laboratories). After DAB (3, 3’-diaminobenzidine) colorimetric development using a Harris’ hematoxylin counterstain, the sections were washed in acid alcohol and Scott’s tap water substitute, dehydrated, and mounted with ClearVue mountant (ThermoScientific). For quantification, after Polaris multispectral analysis (PerkinElmer), 6–7 10x ROIs per tissue were analyzed with InForm (AKOYA Biosciences) automated image analysis software which allows designing an algorithm based on the presence of the positive cells expressed as a percentage on the total cells number (hematoxylin staining was used for the counterstaining).

### TUNEL staining

For in situ cell death detection, four micron sections of FFPE block slides were dewaxed and rehydrated before proteinase K antigen retrieval. Slides were incubated with TUNEL reaction mixture (Roche) 60 min at 37 °C in the dark, following manufacturer’s instruction. After DAPI (SIGMA ALDRICH, 1 µg/ml, 10 min) incubation, slides were mounted in Fluoromount-G (Invitrogen) and analyzed under a fluorescence microscope Nikon Eclipse Ni.

### Serum chemistry

Serum was isolated from whole blood after centrifugations at 1500 × *g* and 14,000 × *g* for 5 min each. Serum chemistry was performed by measurement of ALP, ALT, AST, LDH, glucose, cholesterol—total, triglycerides, creatinine, urea, total protein, albumin, and bilirubin utilizing a commercial kit (Alpha Laboratories Ltd). All kits were adapted for use on a Cobas Fara centrifugal analyzer (Roche Diagnostics Ltd). For all assays, intra-run precision was CV < 4%.

### Serum cytokines

Cytokines (TNF-α, IFN-γ, IL-1β, IL-2, IL-5, KG/CRO, IL-6, IL-10) were quantified in serum using a V-plex Pro-inflammatory Panel kit (Meso Scale Discovery) following the manufacturer’s instructions. Markers such as IL-4, IL-6, IL-10, IL-12p70, IL-13, TNFα, MDC, TARC, Eotaxin2, M-CSF were quantified in cells culture supernatant using U-Plex Panel kit (Meso Scale Discovery) following the manufacturer’s instructions. The plate was read on a QuickPlex SQ 120 analyzer (Meso Scale Discovery). Standards were assayed in duplicate and samples in singlet as recommended.

### Hematology

15 μl of blood from mouse exsanguination was harvested in Microvette CB300 tubes containing EDTA. Samples were analyzed using the Celltac α MEK-6500K instrument, following manufacturer’s instruction, for: Erythrocyte count, Granulocyte count, Hematocrit, Hemoglobin, Lymphocyte count, Mean cell hemoglobin, Mean cell hemoglobin concentration, Mean cell volume, Monocyte count, Platelet count, and Total leukocyte count.

### Statistics

Statistical analyses were performed using GraphPad Prism 9.0 software (GraphPad, La Jolla, CA). The data were analyzed to determine whether they follow a parametric or non-parametric distribution. If the data are normally distributed and have equal variance, a one-way analysis of variance (ANOVA) will be used to determine if there are any significant differences between the groups. One-way ANOVA with Brown-Forsythe and Welch multiple comparisons correction was performed to analyze for changes across time points within the same organ. If the data are not normally distributed, a non-parametric analysis such as the Kruskal–Wallis test, followed by Dunn’s post-hoc test, will be used to compare with the control. A *p* value of <0.05 was considered significant.

### Study approval

All animal experiments were undertaken in accordance with criteria outlined in a license granted under the Animals (Scientific Procedures) Act 1986 and approved by the University of Edinburgh Animal Ethics Committee. This study was conducted in compliance with all relevant ethical regulations, including the principles outlined in the Declaration of Helsinki. The use of human material was granted by the South East Scotland Research Ethics Committee 02, and the use of buffy coats was covered by the Scottish National Blood Transfusion Service (SNBTS). Buffy coats from informed consenting healthy volunteers were obtained in collaboration with SNBTS Blood Donor Center, Edinburgh, United Kingdom, under SNBTS Sample Governance 16-09.

## Supplementary information

supplementary

## Data Availability

The authors declare that all data supporting the findings of this study are available within the paper and its supplementary information files. Further data may be available with permission of corresponding author. The RNA-seq data associated with this study has been deposited in NCBI Gene Expression Omnibus (accession number GSE280475).
