# Supplementary material for: Cryopreserved human alternatively activated macrophages promote resolution of acetaminophen-induced liver injury in mouse
Source: NPJ Regen Med. 2025 Jan 22;10:5. doi: 10.1038/s41536-025-00393-3 (PMC11754469; doi:10.1038/s41536-025-00393-3)
Supplement: Supplementary file 1 — supplementary [file 41536_2025_393_MOESM1_ESM.pdf]

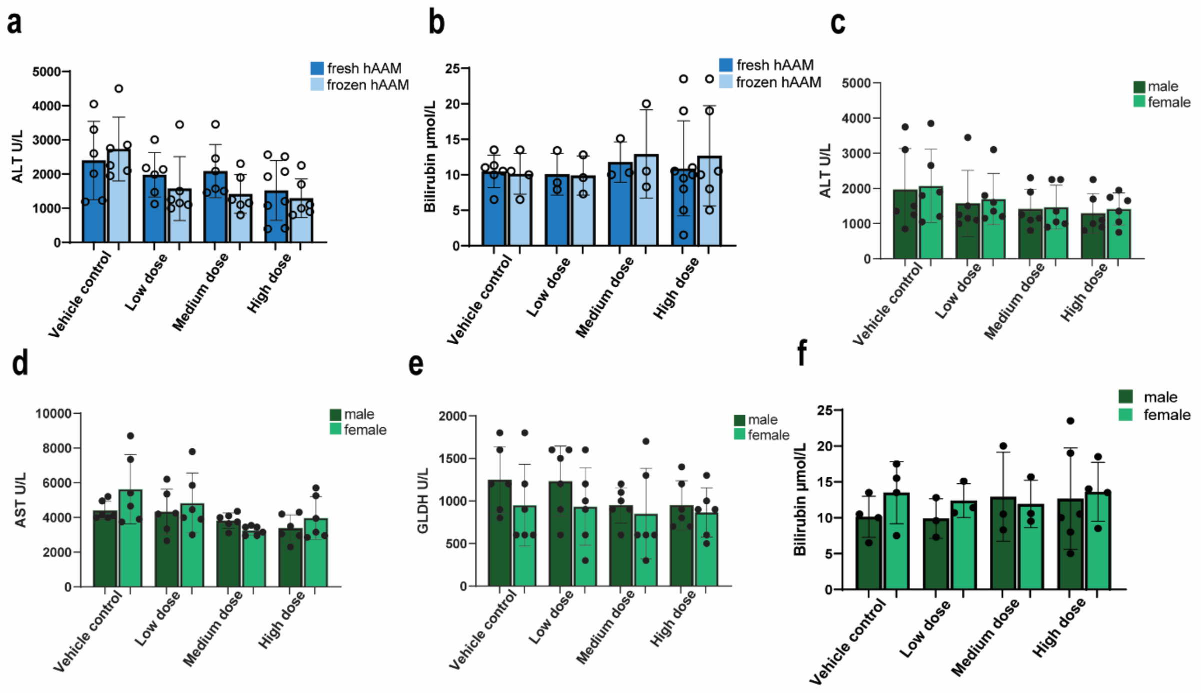

Supplementary Fig. 1. APAP-ALI mice receiving indicated treatments of fresh and thawed hAAM (low dose: 0.25x10^6, medium dose 0.5x10^6 and high dose 1x10^6) at 16 hours after APAP injury. Blood samples were collected after 16 hours, and serum samples were analysed for ALT **(a)** and bilirubin (**b**). Both male and female APAP-ALI mice received indicated treatments of thawed hAAM at 16 hours after APAP injury. After 16 hours the blood samples were collected, and serum samples analyzed for ALT **(c)**, AST **(d),** GLDH **(e)** and bilirubin **(f)** activity. Mean ± SD, n = 3-6; comparison was done with one-way ANOVA test or pot-hoc test, significant diff. among means (P < 0.05).

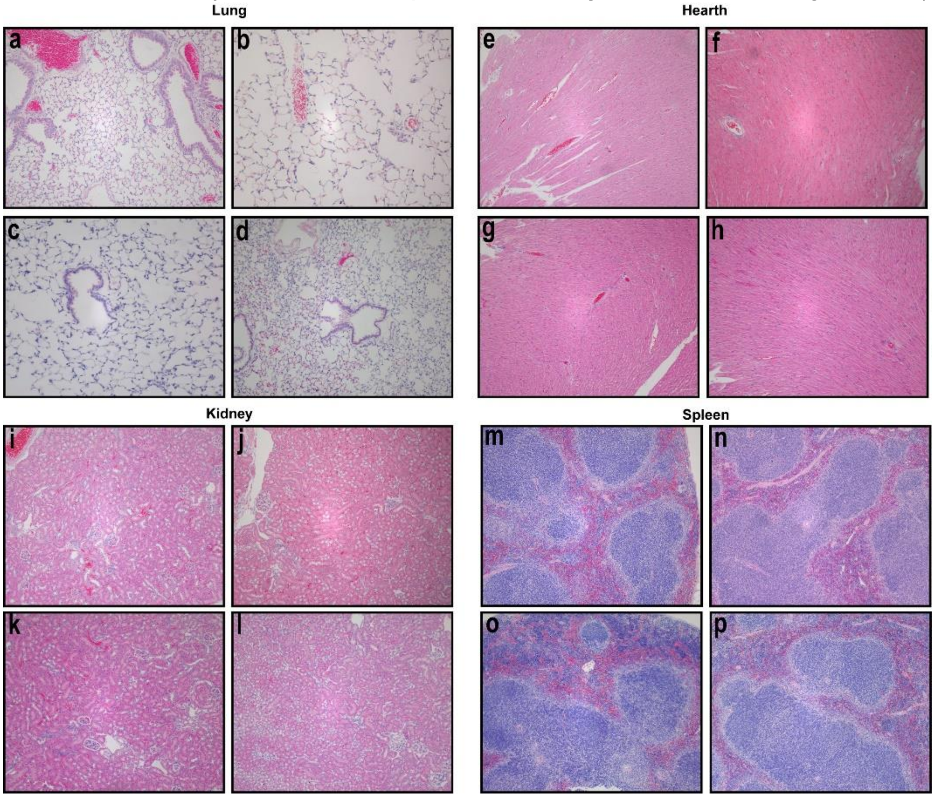

Supplementary Fig. 2: Representative H&E staining of lung (**a-d**), heart (**e-h**), kidney (**i-l**), spleen (**m-p**) and 3 different doses of thawed hAAMs injected in healthy mice for 24 hours: low dose: 1x10^6 (**b, f, j** and **n**), medium dose: 2x10^6 (**c, g, k** and **o**), high dose: 3x10^6 (**d, h, l** and **p**). (**a, e, i** and **m**) vehicle control.

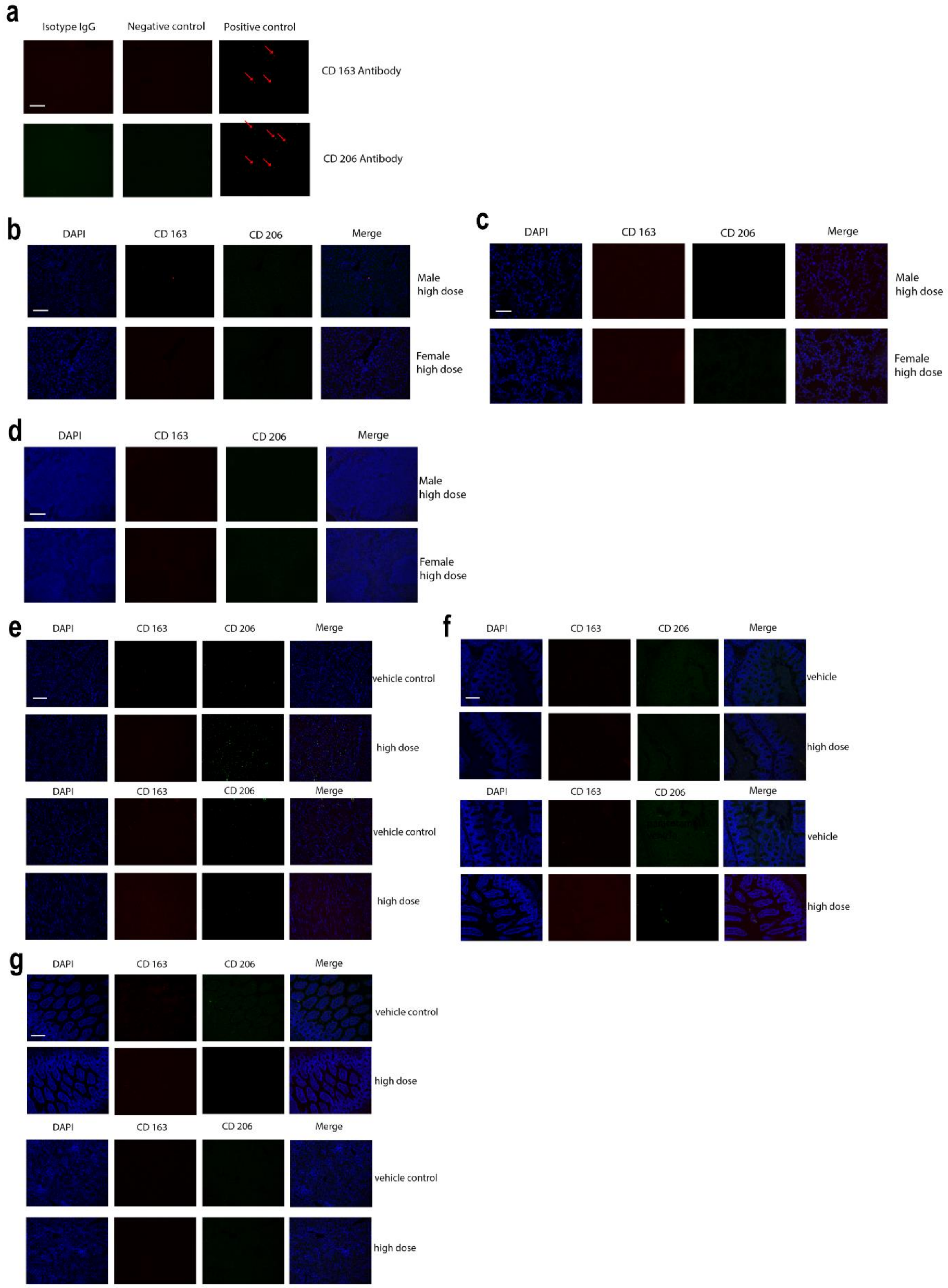

Supplementary Fig. 3: a Representative staining using CD163 and CD206 primary antibody on liver fixed sections. First colon Isotype IgG control, second colon negative control and third colon positive control for CD163 and CD206 antibodies. **b** Representative double staining of CD163 (red), CD206 (green) and nuclei (blue) in liver tissue of APAP-ALI male (top panel) and female (bottom panel) mice at 2 weeks after vehicle control or hAAMs (high dose: 1x10^6) administration. Scale bars — 100 μm. **c** Representative double staining of CD163 (red), CD206 (green) and nuclei (blue) in lung tissue of APAP-ALI male (top panel) and female (bottom panel) mice at 2 weeks after vehicle control or hAAMs (high dose: 1x10^6) administration. Scale bars — 100 μm. **d** Representative double staining of CD163 (red), CD206 (green) and nuclei (blue) in spleen tissue of APAP-ALI male (top panel) and female (bottom panel) mice at 2 weeks after vehicle control or hAAMs (high dose: 1x10^6) administration. Scale bars — 100 μm. **(e-g**) Representative double staining of CD163 (red), CD206 (green) and nuclei (blue) in heart, intestine and kidney tissue respectively of APAP-ALI male (top panel) and female (bottom panel) mice at 2 weeks after vehicle control or hAAMs (high dose: 1x10^6) administration. Scale bars — 100 μm.

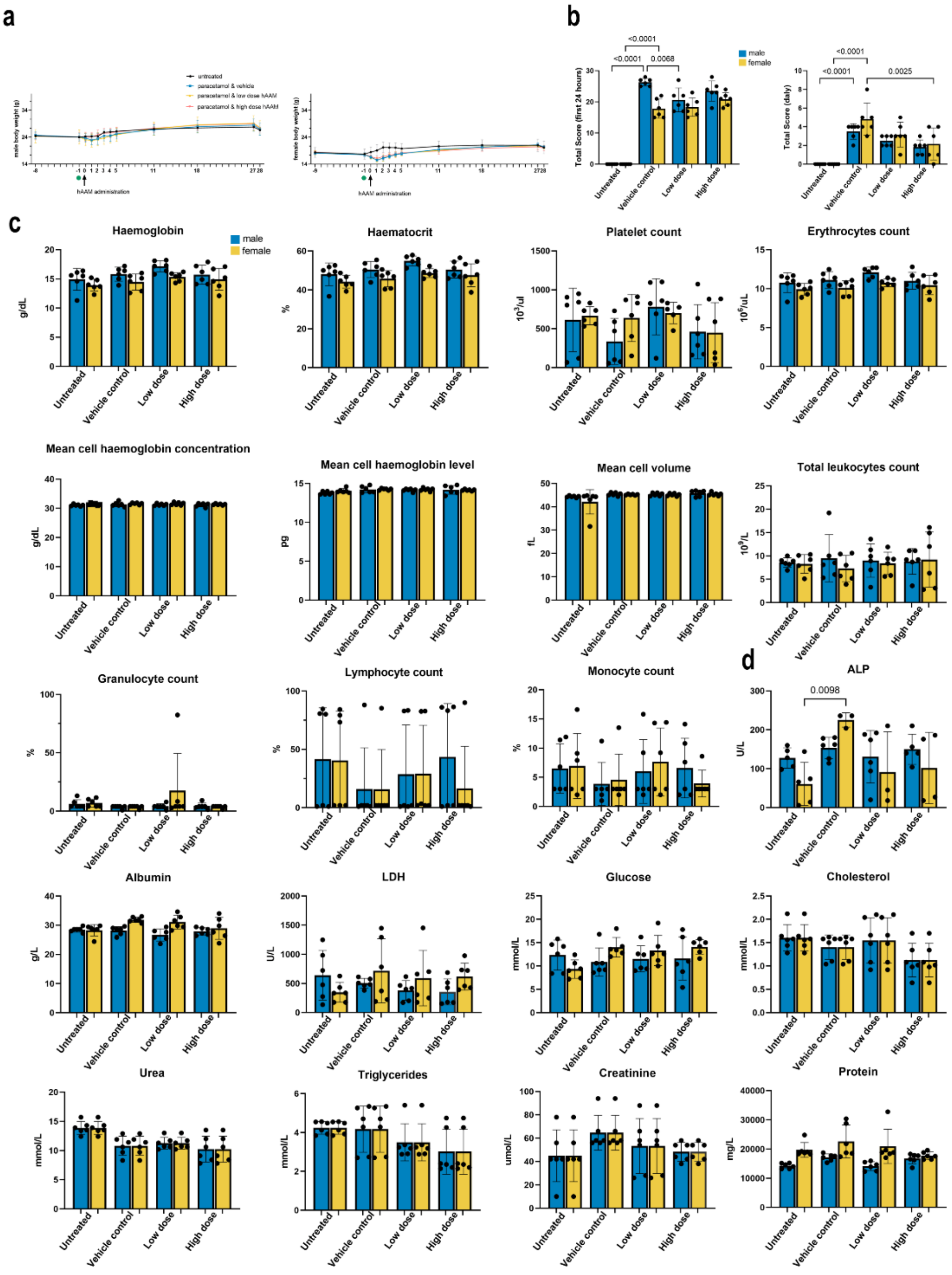

Supplementary Fig. 4: a Body weight of male (left panel) and female (right panel) mice measured pre- and post hAAMs administration in all four groups. APAP was administrated on day -1 (green dot), and hAAMs on day 0 (black arrow). Untreated: study control, APAP treatment and: vehicle control, low (0.5x106) and high (1x10^6) dose of hAAMs. Mean ± SD, n = 6. **b** Total clinical observations score of males and females, measured during the first 24 hours (left panel) and daily for 28 days after hAAMs administration (right panel). Mice were assessed on five parameters (hunching, piloerection, neurological symptoms, responsiveness to touch, skin paleness, and breathing), with a score ranging from 0 to 3 (see table 1); the total score represents the cumulative sum of all scores. Untreated: study control, APAP treatment and vehicle: vehicle control, APAP, and low dose hAAMs: disease induction plus 0.5x10x6 hAAMs, APAP and high dose hAAMs: disease induction plus 1x10^6 hAAMs. Mean ± SD, n = 6. **c** Indicated hematological analysis of ten µL of blood in EDTA in male and female, measured after 4 weeks hAAMs administration. Untreated: study control, APAP treatment and vehicle: vehicle control, APAP, and low dose hAAMs: disease induction plus 0.5x10^6 hAAMs, APAP and high dose hAAMs: disease induction plus 1x10^6 hAAMs. Mean ± SD, n = 6; Comparison was done with one-way ANOVA test or pot-hoc test, significant diff. among means (P < 0.05).

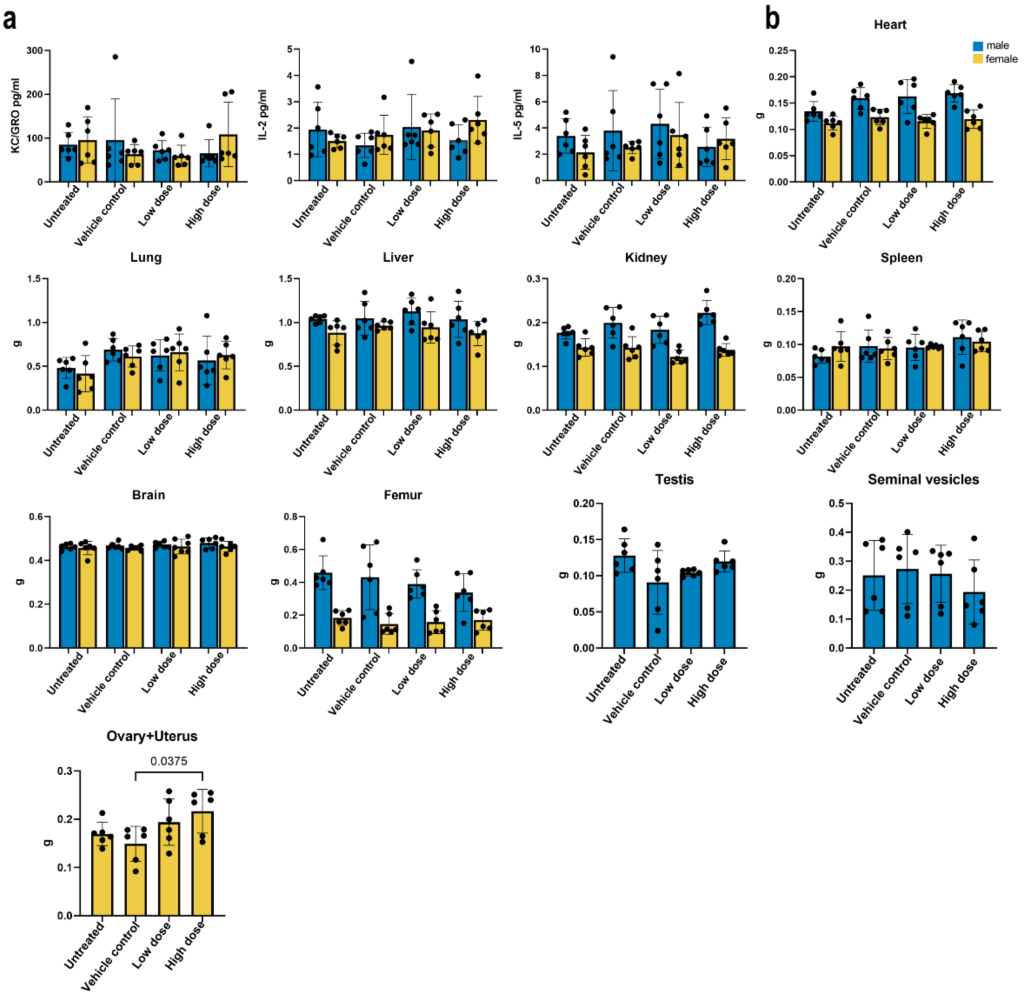

Supplementary Fig. 5: a Serum concentration of indicated proinflammatory cytokines measured in male and female mice at 4 weeks after hAAMs administration. Untreated: study control, APAP treatment and vehicle: vehicle control, APAP, and low dose hAAMs: disease induction plus 0.5x10^6 hAAMs, APAP and high dose hAAMs: disease induction plus 1x10^6 hAAMs. Mean ± SD, n = 6. **b** Organ weight of male and female mice, measured at 4 weeks after hAAMs administration. Untreated: study control, APAP treatment and vehicle: vehicle control, APAP, and low dose hAAMs: disease induction plus 0.5x10^6 hAAMs, APAP and high dose hAAMs: disease induction plus 1x10^6 hAAMs. Mean ± SD, n = 6. Comparison was done with one-way ANOVA test or pot-hoc test, significant diff. among means (P < 0.05).

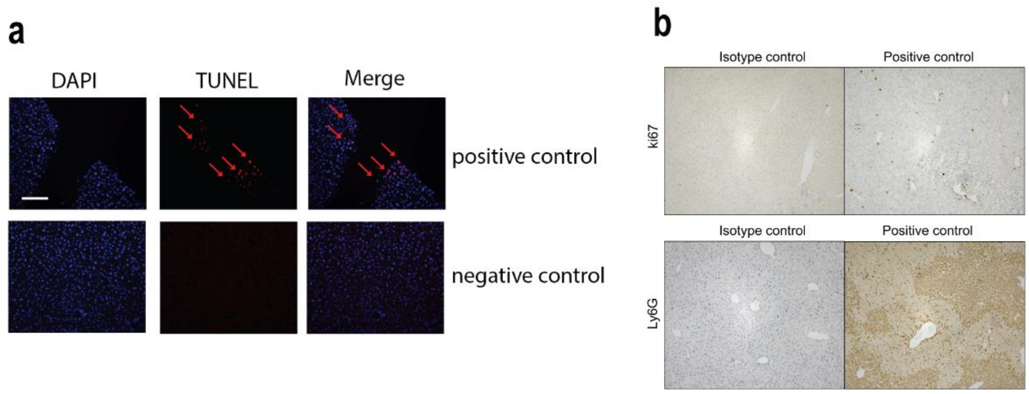

Supplementary Fig. 6: a positive (DNase I recombinant treated) and negative (only Label solution instead of Tunel mixture) controls in liver tissues. **b** Isotype and positive control for Ki67 (up panel) and Ly6G (down panel) staining in mouse liver tissue.

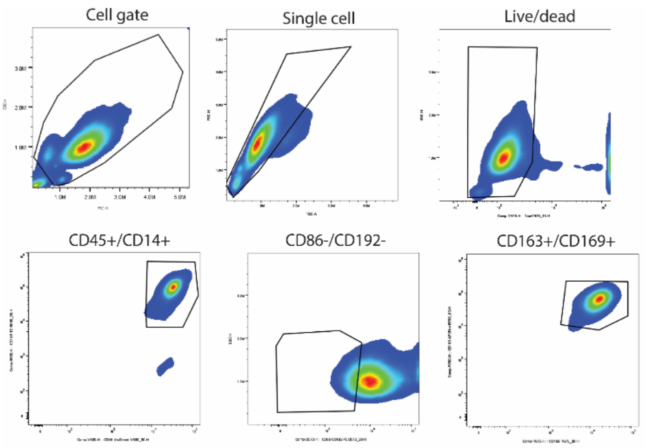

Supplementary Fig. 7: Gating strategy for fresh and cryopreserved hAAMs. Top panels show all cells used for analysis, exclusion of doublets, and selection of live cells. Sub-populations were expressed as proportions of total CD45+/CD14+ cells. hAAMs were negative for CD86/CD192 and positive for CD163/CD169.

Supplementary Table 1

Hunching	SCORE	Skin (paw/ear) paleness	SCORE
Not hunched	0	Absent	0
Mild hunching	1	Mild pallor	1
Moderate hunching	2	Moderate pallor	2
Very hunched	3	Very pale	3
**Responsiveness to touch**		**Neurological symptoms**	
Normal	0	Normal	0
Responds to interaction but slow	1	Slow walk	1
Response delayed and slow	2	Impaired ability to walk straight	2
Unresponsive	3	Lethargic, immobile, no recovery from the supine position when placed by user	3
**Piloerection**		**Breathing**	
Not present	0	Normal	0
Mild piloerection	1	Mild respiratory effort	1
Moderate piloerection	2	Moderate respiratory effort	2
Very pronounced piloerection	3	Very laboured breathing	3
